# Epigenetics of human cutaneous melanoma: setting the stage for new therapeutic strategies

**DOI:** 10.1186/1479-5876-8-56

**Published:** 2010-06-11

**Authors:** Luca Sigalotti, Alessia Covre, Elisabetta Fratta, Giulia Parisi, Francesca Colizzi, Aurora Rizzo, Riccardo Danielli, Hugues JM Nicolay, Sandra Coral, Michele Maio

**Affiliations:** 1 Cancer Bioimmunotherapy Unit, Centro di Riferimento Oncologico, Istituto di Ricovero e Cura a Carattere Scientifico, Via F. Gallini 2, 33081 Aviano, Italy; 2 Division of Medical Oncology and Immunotherapy, Department of Oncology, University Hospital of Siena, Istituto Toscano Tumori, Strada delle Scotte 14, 53100 Siena, Italy

## Abstract

Cutaneous melanoma is a very aggressive neoplasia of melanocytic origin with constantly growing incidence and mortality rates world-wide. Epigenetic modifications (i.e., alterations of genomic DNA methylation patterns, of post-translational modifications of histones, and of microRNA profiles) have been recently identified as playing an important role in melanoma development and progression by affecting key cellular pathways such as cell cycle regulation, cell signalling, differentiation, DNA repair, apoptosis, invasion and immune recognition. In this scenario, pharmacologic inhibition of DNA methyltransferases and/or of histone deacetylases were demonstrated to efficiently restore the expression of aberrantly-silenced genes, thus re-establishing pathway functions. In light of the pleiotropic activities of epigenetic drugs, their use alone or in combination therapies is being strongly suggested, and a particular clinical benefit might be expected from their synergistic activities with chemo-, radio-, and immuno-therapeutic approaches in melanoma patients. On this path, an important improvement would possibly derive from the development of new generation epigenetic drugs characterized by much reduced systemic toxicities, higher bioavailability, and more specific epigenetic effects.

## Introduction

Cutaneous melanoma (CM) is a highly aggressive malignancy originating from melanocytes, which is characterized by constantly growing incidence and mortality rates world-wide [1]. Unlike the majority of human cancers, CM is frequently diagnosed in young and middle-aged adults [2]. Despite representing only 3% of all skin malignancies, CM is responsible for 65% of skin malignancy-related deaths, and the 5-year survival of metastatic CM patients is 7-19% [3,4].

The increasing incidence and the poor prognosis of CM, along with the substantial unresponsiveness of advanced disease to conventional therapies, have prompted significant efforts in defining the molecular alterations that accompany the malignant transformation of melanocytes, identifying epigenetic modifications as important players [5]. "Epigenetics" refers to heritable alterations in gene expression that are not achieved through changes in the primary sequence of genomic DNA. In this respect, the most extensively characterized mediators of epigenetic inheritance are the methylation of genomic DNA in the context of CpG dinucleotides, and the post-translational modifications of core histone proteins involved in the packing of DNA into chromatin [6]. Despite not yet having been extensively characterized, also microRNAs (miRNAs) are emerging as important factors in epigenetic determination of gene expression fate in CM [7].

**Figure 1:**
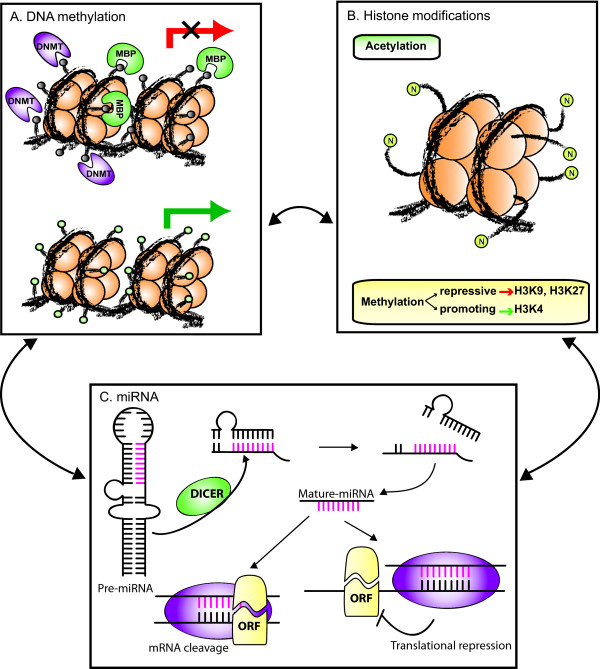
**Epigenetic alterations in CM**. Epigenetic regulation of gene expression involves the interplay of DNA methylation, histone modifications and miRNAs. **A**. Transcriptionally inactive genes (crossed red arrow) are characterized by the presence of methylated cytosines within CpG dinucleotides (grey circles), which is carried out and sustained by DNA methyltransferases (DNMT). Transcriptional repression may directly derive from methylated recognition sequence preventing the binding of transcription factors, or may be a consequence of the binding of methyl-CpG-binding proteins (MBP), which recruit chromatin remodelling co-repressor complexes. Transcriptionally active genes (green arrow) contain demethylated CpG dinucleotides (green circles), which prevent the binding of MBP and co-repressor complexes, and are occupied by complexes including transcription factors and co-activators. **B**. Histones are subjected to a variety of post-translational modifications on their amino terminus (N), including methylation and acetylation, which determine chromatin structure, resulting in the modulation of accessibility of DNA for the transcriptional machinery. The acetylation status of histones is controlled by the balanced action of histone acetyltransferases and histone deacetylases, and acetylated histones have been associated with actively expressed genes. Histone methylation may have both repressive (H3K9, H3K27) or promoting (H3K4) effects on transcription, depending on which residue is modified. **C**. MiRNAs are small non-coding RNAs that regulate the expression of complementary mRNAs. Once incorporated into the RNA-induced silencing complex, miRNAs recognize their target mRNA through a perfect or nearly perfect sequence complementarity, and direct their endonucleolytic cleavage or inhibit their translation. DICER, RNase III family endoribonuclease, ORF, open reading frame.

DNA methylation occurs at the C5 position of cytosine in the context of CpG dinucleotides and it is carried out by different DNA methyltransferases (DNMT) that have distinct substrate specificities: DNMT1 preferentially methylates hemimethylated DNA and has been associated with the maintenance of DNA methylation patterns [8]; DNMT3a and 3b do not show preference for hemimethylated DNA and have been implicated in the generation of new methylation patterns [9,10]. Besides this initial strict categorization, recent evidences are indicating that all three DNMTs may possess both *de novo *and maintenance functions *in vivo*, and that they cooperate in establishing and maintaining DNA methylation patterns [11-14]. The methylation of promoter regions inhibits gene expression either by directly blocking the binding of transcriptional activators or by binding methyl-CpG-binding domain (MBD) proteins that silence gene expression through the recruitment of chromatin remodeling co-repressor complexes (Figure 1) [15,16].

Genomic DNA in the nucleus is packed into the chromatin, the base unit of which is the nucleosome: a histone octamer core comprising two copies each of histones H2A, H2B, H3 and H4, around which about 147 bp of DNA are wrapped. Each histone contains flexible N-terminal tails protruding from the nucleosomes, which are extensively targeted by post-translational modifications, including acetylation and methylation. These modifications determine how tightly the chromatin is compacted, thus playing a central regulatory role in gene expression. The acetylation status of histones is controlled by the balanced action of histone acetyltransferases and histone deacetylases (HDAC), and acetylated histones have been associated with actively expressed genes. On the other hand, methylation of histones, accomplished by histone methyl transferases (HMT), may have both repressive (H3 lysine (K) 9, H3K27) or promoting (H3K4) effects on transcription, depending on the target residue (Figure 1) [17]. Histone modifications comprehensively define the so called "histone code" that is read by multi-protein chromatin remodelling complexes to finally determine the transcriptional status of the target gene by modulating chromatin compaction grade [18].

MiRNAs, the most recently discovered mediators of epigenetic gene regulation, are endogenous non-coding RNA about 22 nucleotide long. MiRNAs are transcribed in the nucleus by RNA polymerase II into long primary transcripts (pri-miRNAs), which are further processed by a complex of the RNase III Drosha and its cofactor DGCR8 into the about 60 nucleotides long precursor miRNAs (pre-miRNAs). Pre-miRNAs are subsequently exported to the cytoplasm where the RNase III Dicer cuts off the loop portion of the stem-loop structure, thus reducing pre-miRNAs to short double strands. Finally, each pre-miRNA is unwound by a helicase into the functional miRNA. Once incorporated into the RNA-induced silencing complex, miRNAs recognize their target mRNA through a perfect or nearly perfect sequence complementarity, and direct their endonucleolytic cleavage or inhibit their translation (Figure 1). Each miRNA is predicted to have many targets, and each mRNA may be regulated by more than one miRNA [7].

Rather than acting separately, the above described epigenetic regulators just represent different facets of an integrated apparatus of epigenetic gene regulation (Figure 1). Indeed, recent studies showed that DNA methylation affects histone modifications and *vice versa*, to make up a highly complex epigenetic control mechanism that cooperates and interacts in establishing and maintaining the patterns of gene expression [19]. Along this line, miRNA were demonstrated to be target of regulation by DNA methylation, while concomitantly being able to regulate the expression of different chromatin-modifying enzymes [7].

### Identifying epigenetic alterations in CM

The maintenance of epigenetic marks, either natural or acquired through neoplastic transformation, requires the function of specific enzymes, such as DNMT and HDAC. The pharmacologic and/or genetic inactivation of DNMT and/or HDAC erases these epigenetic marks, leading to the reactivation of epigenetically-silenced genes [20]. This pharmacologic reversal has been widely exploited to identify genes and cellular pathways that were potentially inactivated by aberrant epigenetic alterations in CM [21,22]: genes down-regulated in CM as compared to melanocytes, and whose expression was induced/up-regulated by epigenetic drugs, were assumed to be epigenetically inactivated in CM. Gene expression microarrays were recently used to assess the modulation of the whole transcriptome by the DNMT inhibitor 5-aza-2'-deoxycytidine (5-AZA-CdR) in different CM cell lines, allowing to identify a large number of genes that were potentially inactivated by promoter methylation in CM, as further supported by preliminary methylation analyses performed on 20 CM tissues [21]. A similar approach investigated genome-wide gene re-expression/up-regulation following combined treatment with 5-AZA-CdR and the HDAC inhibitor (HDACi) Trichostatin A (TSA), to identify genes suppressed in CM cells by aberrant promoter hypermethylation and histone hypoacetylation [22]. Despite the power of these approaches, care must be taken to correctly interpret these high-throughput results [23]: an adequate statistical treatment of data is mandatory to obtain robust findings, which are finally required to be validated through the direct evaluation of the correlation between promoter methylation or histone post-translational modifications and the expression of the identified genes, in large cohorts of CM lesions. Along this line, the specific functional role of each of these genes in CM biology is being further examined either by gene transfer or RNA interference approaches in CM cell lines [21].

The direct evaluation of the DNA methylation status of the genes of interest is performed through different technologies that usually rely on the modification of genomic DNA with sodium bisulfite, which converts unmethylated, but not methylated, cytosines to uracil, allowing methylation data to be read as sequence data [24,25]. The most widely used bisulfite-based methylation assays are: i) bisulfite sequencing [25]; ii) bisulfite pyrosequencing [26]; iii) Combined Bisulfite Restriction Analysis (CoBRA) [27]; iv) Methylation-Specific PCR (MSP) [28]; v) MSP real-time PCR [29]. Global genomic DNA methylation assays may be used to directly assess the overall role of aberrant DNA methylation in CM biology, and include: i) methylation of the repetitive elements *LINE-1 *and *Alu *by CoBRA or pyrosequencing [30]; ii) 5-methyl-cytosine content by HPLC or capillary electrophoresis [31]; iii) whole genome evaluation of CpG island methylation by CpG island microarrays [32]. Along this line, a genome-wide integrative analysis of promoter methylation and gene expression microarray data might assist in the identification of methylation markers that are likely to have a biologic relevance due to their association with altered levels of expression of the respective gene [32]. The bias posed by the pre-definition of the sequences to be investigated, which is inherently associated with CpG island microarray analyses, will be most likely overcome in the next few years by exploiting the next-generation sequencing technologies [33]. The application of these approaches on genomic DNA that has been enriched in methylated sequences by affinity chromatography, with either anti-5-methyl-cytosine antibodies or MBD proteins, can be expected to provide a detailed and essentially unbiased map of the whole methylome of CM.

On the other hand, global levels of histone modifications can be evaluated through either mass spectrometry or Western blot analysis [34]. The direct evaluation of gene-associated histone post-translational modifications relies on immunoprecipitation of chromatin with antibodies specifically recognizing histones with modified tails, followed by PCR amplification of the gene of interest. This immunoprecipitation approach might be eventually coupled to genomic microarray hybridization or next-generation sequencing to examine at whole genome level the aberrant genetic patterns of histone post-translational modifications [35].

### DNA methylation

Neoplastic transformation is accompanied by a complex deregulation of the cellular DNA methylation homeostasis, resulting in both gene-specific hypermethylation and genome-wide hypomethylation [6].

**Figure 2:**
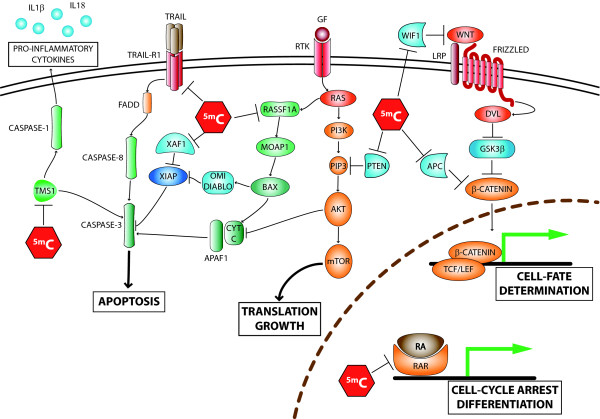
**Selected pathways altered by DNA hypermethyation in CM**. Aberrant promoter hypermethylation in CM may suppress the expression of APC, PTEN, RASSF1A, TMS1, TRAIL-R1, XAF1, and WIF1, leading to deregulation of different pathways, including apoptosis, cell cycle, cell-fate determination, cell growth, and inflammation. Gene symbol: APAF1, apoptotic peptidase activating factor 1; APC, adenomatous polyposis coli; BAX, BCL2-associated X protein; CYT C, cytochrome C; DIABLO, direct IAP-binding protein with low pI; DVL, dishevelled; FADD, Fas-associating protein with death domain; GF, Growth Factor; GSK3β, glycogen synthase kinase 3 beta; IL, interleukin; LRP, LDL receptor family; MOAP1, modulator of apoptosis 1; mTOR, mammalian target of rapamycin; PI3K, phosphoinositide-3-kinase; PIP3, phosphatidylinositol (3,4,5)-trisphosphate; PTEN, phosphatase and tensin homolog; RAR, retinoic acid receptor; RASSF1A, Ras association domain family 1; RTK, Receptor Tyrosine Kinase; TCF/LEF, T-cell factor/lymphoid enhancer factor; TMS1, Target Of Methylation Silencing 1; TRAIL, TNF-related apoptosis inducing ligand; TRAIL-R1, TRAIL receptor 1; WIF1, Wnt inhibitory factor 1; XAF1, XIAP associated factor 1; XIAP, X-linked inhibitor of apoptosis.

Aberrant DNA hypermethylation is a frequent event in CM and represents an important mechanism utilized by neoplastic cells to shut off different tumor suppressor genes (TSG) (Figure 2, Table 1). Inactivation by DNA hypermethylation was found to affect also genes that are not typically targeted by gene deletion/mutation, providing complementary tools for melanocyte transformation. Nevertheless, genetic and epigenetic alterations also co-operate to shut off specific gene functions, as it was seen for the *CDKN2A *locus [36,37]. *CDKN2A *can be regarded as the major gene involved in CM pathogenesis and predisposition, being inactivated in the majority of sporadic CM and representing the most frequently mutated gene inherited in familial CM [38]. *CDKN2A *locus encodes two proteins, p16^INK4A ^and p14^ARF^, which exert tumor suppressor functions through the pRB and the p53 pathways, respectively [38]. Recent data have demonstrated that aberrant promoter hypermethylation at *CDKN2A *locus independently affects p16^INK4A ^and p14^ARF^, which are methylated in 27% and 57% of metastatic CM samples, respectively [37]. These epigenetic alterations had an incidence comparable to gene deletions/mutations, and frequently synergized with them to achieve a complete loss of TSG expression: gene deletion eliminating one allele, promoter hypermethylation silencing the remaining one. This combined targeting of the *CDKN2A *locus, through epigenetic and genetic alterations, led to the concomitant inactivation of both p16^INK4A ^and p14^ARF ^in a significant proportion of metastatic CM examined, likely allowing neoplastic cells to evade the growth arrest, apoptosis and senescence programs triggered by the pRB and p53 pathways. Besides specific examples, on the whole, gene-specific hypermethylation has been demonstrated to silence genes involved in all of the key pathways of CM development and progression, including cell cycle regulation, cell signalling, differentiation, DNA repair, apoptosis, invasion and immune recognition (Figure 2, Table 1). *RAR-β2*, which mediates growth arrest, differentiation and apoptotic signals triggered by retinoic acids (RA), together with *RASSF1A*, which promotes apoptosis and growth arrest, and *MGMT*, which is involved in DNA repair, are the most frequent and well-characterized hypermethylated genes in CM, being methylated in 70% [39], 55% [40,41] and 34% of CM lesions, respectively [39] (Figure 2, Table 1). Notably, a very high incidence of promoter methylation has been observed for genes involved in the metabolic activation of chemotherapeutic drugs (i.e., *CYP1B1*, methylated in 100% CM lesions [21], and *DNAJC15*, methylated in 50% CM lesions [21]), which might contribute, together with the impairment of the apoptotic pathways, to the well-known resistance of CM cells to conventional chemotherapy. The list of genes hypermethylated in CM is continuously expanding, and it is including new genes that are hypermethylated in virtually all CM lesions examined (e.g., *QPCT*, methylated in 100% CM [21]; *LXN*, methylated in 95% CM [21]), though their function/role in CM progression has still to be addressed. Interestingly, some genes, such as *RAR-β2*, are found methylated with similar frequencies in primary and metastatic CM, suggesting their methylation as being an early event in CM, while others have higher frequencies in advanced disease (e.g., *MGMT, RASSF1A, DAPK*), suggesting the implication of their aberrant hypermethylation in CM progression [39]. Along this line, a recent paper by Tanemura *et al *reported the presence of a CpG island methylator phenotype (i.e., high incidence of concomitant methylation of different CpG islands) in CM, which was associated with advancing clinical tumor stage. In particular, the TSG *WIF1,TFPI2*, *RASSF1A*, and *SOCS1*, and the methylated in tumors (*MINT*) loci *17 *and *31*, showed a statistically significant higher frequency of methylation from AJCC stage I to stage IV tumors [42].

**Table 1:** Genes with an altered DNA methylation status in human CM

PATHWAY	GENE	METHYLATION STATUS IN CM^a^	PERCENT	FREQUENCY	SOURCE	MODULATED BY 5-AZA-CdR	REF.
							
APOPTOSIS	*DAPK^b^*	methylated	19	16/86	tumor	ND^c^	[39]
	*HSPB6*	methylated	100	8/8	cell line	YES	[32]
	*HSPB8*	methylated	69	11/16	tumor	YES	[128]
	*RASSF1A*	methylated	NA	NA	cell line	YES	[41]
		methylated	46	6/13	cell line	YES	[129]
		methylated	69	11/16	cell line	ND	[44]
		methylated	63	26/41	serum	NA	[130]
		methylated	28	13/47	serum	NA	[124]
		methylated	19	6/31	serum	NA	[39]
		methylated	25	10/40	tumor	ND	[101]
		methylated	36	9/24	tumor	NA	[129]
		methylated	55	24/44	tumor	YES	[40]
		methylated	57	49/86	tumor	YES	[39]
	*TMS1*	methylated	8	3/40	tumor	ND	[101]
		methylated	50	5/10	tumor	YES	[131]
	*TNFRSF10C*	methylated	57	23/40	tumor	YES	[101]
	*TNFRSF10D*	methylated	80	32/40	tumor	YES	[101]
	*TP53INP1*	methylated	19	3/16	tumor	YES	[22]
	*TRAILR1*	methylated	80	8/10	cell line	YES	[98]
		methylated	13	5/40	tumor	ND	[101]
	*XAF1*	methylated	NA	NA	cell line	YES	[99]
							

ANCHORAGE-INDEPENDENT GROWTH	*TPM1*	methylated	8	3/40	tumor	ND	[101]
							

CELL CYCLE	*CDKN1B*	methylated	0	0/13	cell line	ND	[129]
		methylated	0	0/40	tumor	ND	[101]
		methylated	9	4/45	tumor	ND	[132]
	*CDKN1C*	methylated	35	7/20	tumor	YES	[21]
	*CDKN2A*	methylated	76	31/41	serum	NA	[130]
		methylated	10	3/30	tumor	YES	[36]
		methylated	13	5/40	tumor	ND	[101]
		methylated	19	11/59	tumor	ND	[133]
		methylated	57	34/60	tumor	ND	[37]
	*TSPY*	methylated	100	5/5 male patients	tumor and cell line	YES	[134]
							

CELL FATE DETERMINATION	*MIB2*	methylated	19	6/31	tumor	ND	[135]
	*APC*	methylated	15	6/40	tumor	ND	[101]
		methylated	17	9/54	tumor	YES	[136]
	*WIF1*	methylated	NA	NA	cell line	YES	[137]
							

CHROMATIN REMODELING	*NPM2*	methylated	50	12/24	tumor	YES	[32]
							

DEGRADATION OF MISFOLDED PROTEINS	*DERL3*	methylated	23	3/13	cell line	NO	[138]
							

DIFFERENTIATION	*ENC1*	methylated	6	1/16	tumor	YES	[22]
	*GDF15*	methylated	75	15/20	tumor	YES	[21]
	*HOXB13*	methylated	20	4/20	tumor	YES	[21]
							

DNA REPAIR	*MGMT*	methylated	0	0/13	cell line	ND	[129]
		methylated	50	8/16	cell line	ND	[44]
		methylated	63	26/41	serum	NA	[130]
		methylated	19	6/31	serum	NA	[39]
		methylated	13	5/40	tumor	ND	[101]
		methylated	31	26/84	tumor	ND	[139]
		methylated	34	29/86	tumor	YES	[39]
							

DRUG METABOLISM	*CYP1B1*	methylated	100	20/20	tumor	YES	[21]
	*DNAJC15*	methylated	50	10/20	tumor	YES	[21]
							

EXTRACELLULAR MATRIX	*COL1A2*	methylated	63	45/24	tumor	YES	[32]
		methylated	80	16/20	tumor	YES	[21]
	*MFAP2*	methylated	30	6/20	tumor	YES	[21]
							

IMMUNE RECOGNITION	*BAGE*	demethylated	83	10/12	cell line	YES	[140]
	*HLA class I*	methylated	NA	NA	cell line	YES	[97]
	*HMW-MAA*	methylated	NA	NA	tumor and cell line	YES	[93]
	*MAGE-A1*	demethylated	NA	NA	cell line	YES	[45]
	*MAGE-A2, -A3, -A4*	demethylated	NA	NA	tumor	YES	[47]
							

INFLAMMATION	*PTGS2*	methylated	20	4/20	tumor	YES	[21]
							

INVASION/METASTASIS	*CCR7*	no CpG island	NA	NA	cell line	YES	[141]
	*CDH1*	methylated	88	14/16	cell line	ND	[44]
	*CDH8*	methylated	10	2/20	tumor	YES	[21]
	*CDH13*	methylated	44	7/16	cell line	ND	[44]
	*CXCR4*	methylated	NA	NA	cell line	YES	[141]
	*DPPIV*	methylated	80	8/10	cell line	YES	[142]
	*EPB41L3*	methylated	5	1/20	tumor	YES	[21]
	*SERPINB5*	methylated	100	7/7	cell line	ND	[143]
		methylated	13	5/40	tumor	YES	[144]
	*LOX*	methylated	45	18/40	tumor	YES	[101]
	*SYK*	methylated	3	1/40	tumor	ND	[101]
		methylated	30	6/20	tumor	YES	[21]
	*TFPI-2*	methylated	13	5/40	tumor	ND	[101]
		methylated	29	5/17	tumor	YES	[145]
	*THBD*	methylated	20	8/40	tumor	YES	[101]
		methylated	60	12/20	tumor and cell line	YES	[146]
	*TIMP3*	methylated	13	5/40	tumor	ND	[101]
							

PROLIFERATION	*MT1G*	methylated	21	5/24	tumor	YES	[32]
	*WFDC1*	methylated	20	4/20	tumor	YES	[21]
		methylated	25	10/40	tumor	ND	[101]
							

SIGNALING	*DDIT4L*	methylated	29	7/24	tumor	YES	[32]
	*ERα*	methylated	17	2/12	cell line	ND	[129]
		methylated	50	8/16	cell line	ND	[44]
		methylated	24	26/109	serum	NA	[123]
		methylated	51	55/107	tumor	ND	[123]
	*PGRβ*	methylated	56	9/16	cell line	ND	[44]
	*PRDX2*	methylated	8	3/36	tumor	YES	[138]
	*PTEN*	methylated	23	3/13	cell line	ND	[129]
		methylated	62	23/37	serum	YES	[147]
		methylated	0	0/40	tumor	NA	[101]
	*3-OST-2*	methylated	15	2/13	cell line	ND	[129]
		methylated	56	14/25	tumor	NA	[129]
	*RARRES1*	methylated	13	2/16	tumor	YES	[22]
	*RARβ2*	methylated	44	7/16	cell line	ND	[44]
		methylated	46	6/13	cell line	YES	[129]
		methylated	13	4/31	serum	NA	[39]
		methylated	22	5/23	tumor	NA	[129]
		methylated	20	5/25	tumor	YES	[129]
		methylated	60	24/40	tumor	ND	[101]
		methylated	70	74/106	tumor	YES	[39]
	*RIL*	methylated	88	14/16	cell line	ND	[44]
	*SOCS1*	methylated	75	30/40	tumor	ND	[101]
		methylated	76	31/41	serum	NA	[130]
	*SOCS2*	methylated	44	18/41	serum	NA	[130]
		methylated	75	30/40	tumor	ND	[101]
	*SOCS3*	methylated	60	3/5	tumor	YES	[148]
	*UNC5C*	methylated	23	3/13	cell line	NO	[138]
							

VESCICLE TRANSPORT	*Rab33A*	methylated	100	16/16	tumor and cell line	YES	[149]
							

TRANSCRIPTION	*HAND1*	methylated	15	2/13	cell line	ND	[129]
	*HAND1*	methylated	63	10/16	cell line	ND	[44]
	*OLIG2*	methylated	63	10/16	cell line	ND	[44]
	*NKX2-3*	methylated	63	10/16	cell line	ND	[44]
	*PAX2*	methylated	38	6/16	cell line	ND	[44]
	*PAX7*	methylated	31	5/16	cell line	ND	[44]
	*RUNX3*	methylated	23	3/13	cell line	ND	[129]
		methylated	29	5/17	cell line	ND	[150]
		methylated	4-17	2/52-5/30	tissues	NA	[150]
							

TBD	*BST2*	methylated	50	10/20	tumor	YES	[21]
	*FAM78A*	methylated	8	1/13	cell line	NO	[138]
	*HS3ST2*	methylated	56	14/25	tumor	ND	[129]
	*LRRC2*	methylated	5	1/20	tumor	YES	[21]
	*LXN*	methylated	95	19/20	tumor	YES	[21]
	*PCSK1*	methylated	60	12/20	tumor	YES	[21]
	*PPP1R3C*	methylated	25	4/16	tumor	YES	[22]
	*PTPRG*	methylated	8	1/13	cell line	NO	[138]
	*QPCT*	methylated	100	20/20	tumor	YES	[21]
	*SLC27A3*	methylated	46	6/13	cell line	NO	[138]
							

^a^, methylation status of the gene found in CM as compared to that found in normal tissue;

^b^, gene symbol: APAF-1, Apoptotic Protease Activating Factor 1; APC, adenomatous polyposis coli; BAGE, B melanoma antigen; BST2, bone marrow stromal cell antigen 2; CCR7, chemokine (C-C motif) receptor 7; CDH1, cadherin 1;CDH8, cadherin 8; CDH13, cadherin 13; CDKN1B, cyclin-dependent kinase inhibitor 1B; CDKN1C, cyclin-dependent kinase inhibitor 1C; CDKN2A, cyclin-dependent kinase inhibitor 2A; COL1A2, alpha 2 type I collagen; CXCR4, chemokine (C-X-C motif) receptor 4; CYP1B1, cytochrome P450, family 1, subfamily B, polypeptide 1; DAPK, death-associated protein kinase; DDIT4L, DNA-damage-inducible transcript 4-like; DERL3, Der1-like domain family, member 3; DNAJC15, DnaJ homolog, subfamily C, member 15; DPPIV, dipeptidyl peptidase IV; ENC1, ectodermal-neural cortex-1; EPB41L3, erythrocyte membrane protein band 4.1-like 3; ERα, Estrogen Receptor alpha; FAM78A, Family with sequence similarity 78, member A; GDF15, growth differentiation factor 15; HAND1, heart and neural crest derivatives expressed 1; HLA class I, human leukocyte class I antigen; HMW-MAA, high molecular weight melanoma associated antigen; HOXB13, homeobox B13; HS3ST2, heparan sulfate (glucosamine) 3-O-sulfotransferase 2; HSPB6, heat shock protein, alpha-crystallin-related, B6; HSPB8 heat shock 22 kDa protein 8; LRRC2, leucine rich repeat containing 2; LOX, lysyl oxidase; LXN, latexin; MAGE, melanoma-associated antigen, MFAP2, microfibrillar-associated protein 2; MGMT, O-6-methylguanine-DNA methyltransferase; MIB2, mindbomb homolog 2; MT1G, metallothionein 1G; NKX2-3, NK2 transcription factor related, locus 3; NPM2, nucleophosmin/nucleoplasmin 2; OLIG2, oligodendrocyte lineage transcription factor 2; PAX2, paired box 2; PAX7, paired box 7; PCSK1, proprotein convertase subtilisin/kexin type 1; PGRβ, progesterone receptor β; PPP1R3C, protein phosphatase 1, regulatory (inhibitor) subunit 3C; PRDX2, Peroxiredoxin; PTEN, Phosphatase and tensin homologue; PTGS2, prostaglandin-endoperoxide synthase 2; PTPRG, Protein tyrosine phosphatase, receptor type, G; QPCT, glutaminyl-peptide cyclotransferase; RARB, Retinoid Acid Receptor β2; RASSF1A, RAS associacion domain family 1; RIL, Reversion-induced LIM; RUNX3, runt-related transcription factor 3; SERPINB5, serpin peptidase inhibitor, clade B, member 5; SLC27A3, Solute carrier family 27; SOCS, suppressor of cytokine signaling; SYK, spleen tyrosine kinase; TFPI-2, Tissue factor pathway inhibitor-1; THBD, thrombomodulin; TIMP3, tissue inhibitor of metalloproteinase 3; TMS1, Target Of Methylation Silencing 1; TNFRSF10C, tumor necrosis factor receptor superfamily, member 10C; TNFRSF10D, tumor necrosis factor receptor superfamily, member 10D; TP53INP1, tumor protein p53 inducible nuclear protein 1; TPM1, tropomyosin 1 (alpha); TRAILR1, TNF-related apoptosis inducing ligand receptor 1; TSPY, testis specific protein, Y-linked; UNC5C, Unc-5 homologue C; WFDC1, WAP four-disulfide core domain 1; WIF1, Wnt inhibitory factor 1; XAF1, XIAP associated factor 1.

^c^, NA, not applicable; ND, not done; TBD, to be determined.

Besides TSG hypermethylation, genome-wide hypomethylation might contribute to tumorigenesis and cancer progression by promoting genomic instability, reactivating endogenous parasitic sequences and inducing the expression of oncogenes [43]. In this context, Tellez *et al *measured the level of methylation of the *LINE-1 *and *Alu *repetitive sequences to estimate the genome wide methylation status of CM cell lines [44]. With this approach they were able to demonstrate that CM cell lines do have hypomethylated genomes as compared to melanocytes. Moreover, the extent of repetitive elements hypomethylation inversely correlated with the number of TSG aberrantly inactivated by promoter hypermethylation. The data obtained are particularly interesting since they shed initial light on how the two apparently antithetical phenomena of TSG hypermethylation and global loss of genomic 5-methylcytosine content might be interconnected. In fact, it could be speculated that, upon an initial genome-wide demethylation wave, the cell attempts to re-establish methylation patterns of repetitive elements. This wave of re-methylation could find promoter CpG islands more prone to *de novo *methylation, thus resulting in a more frequent silencing of TSG [44]. On the other hand, a direct association was found between genome-wide demethylation and *de novo *expression of tumor associated antigens belonging to the Cancer Testis Antigens (CTA) family (e.g., *MAGE-A *and *NY-ESO *genes) [45-47]. CTA are not expressed in normal tissues except testis and placenta, while they are expressed with variable frequencies in CM tissues [47]. This characteristic tissue distribution, and their ability to generate both cellular and humoral immune responses, identified CTA as ideal targets for immunotherapy of CM patients, and led to the development of several clinical trials that are providing promising therapeutic results [48]. Recent data demonstrated that the frequently observed intratumoral heterogeneity of CTA expression, which might impair the clinical success of CTA-based immunotherapies, is itself sustained by the intratumoral heterogeneous methylation of their promoters [49]. This promoter methylation heterogeneity is further inherited at single cell level, propagating the heterogeneous CTA expression profile to daughter generations [50]. The reported association between aberrant hypomethylation of CTA promoters and CTA expression has been most recently confirmed also on populations of putative CM stem cells [51], providing further support to the key role of deregulated DNA methylation in CM development and progression, and on the potential of CTA as therapeutic targets in CM [52].

### Histone post-translational modifications

In contrast to the massive information existing on the altered DNA methylation patterns occurring in CM, the data available on aberrant post-translational modifications of histones are comparatively limited and mostly indirect, being frequently just inferred from the modulation of gene expression observed following treatment with pharmacologic inhibitors of histone-modifying enzymes (i.e., HDACi). This essential lack of direct information likely reflects the more challenging approaches that are required for evaluating histone modifications associated to the transcriptional status of specific genes. In this respect, selected issues are: i) the myriad of combinations of post-translational modifications that are possible for each histone; ii) the requirement of chromatin immunoprecipitation approaches with antibodies specific for each histone modification; and, iii) the need of huge amounts of starting DNA, which essentially precluded the evaluation of tumor tissues. These limitations, however, are likely to be overcome soon thanks to the availability of the new generation high-throughput technologies and whole genome amplification protocols.

Despite these restrictions, the available data suggest that aberrant post-translational modifications of histones, and in particular their hypoacetylation, profoundly influence CM cell biology by affecting cell cycle regulation, cell signaling, differentiation, DNA repair, apoptosis, invasion and immune response (Table 2). Among these, the alterations of cell cycle regulation and apoptosis are the better characterized, and mainly involve histone hypoacetylation-mediated down-regulation of CDKN1A/P21, and of the pro-apoptotic proteins APAF-1, BAX, BAK, BID, BIM, caspase 3 and caspase 8 [53-56]. These findings might, to some extent, provide a molecular background for a peculiar characteristic of CM. In fact, CM cells usually express high levels of wild type p53, which represents the master regulator of DNA repair that directs cells to apoptosis in case of DNA repair failure [57]. Despite this, CM cells are extremely resistant to undergoing apoptosis following conventional cytotoxic therapies. In light of the information above, it could be speculated that this behaviour of CM cells could depend, at least in part, on the epigenetic impairment of apoptotic pathways.

**Table 2:** Genes potentially regulated by modifications of histone acetylation in human CM

PATHWAY	GENE	SOURCE	HDACi	MODULATION BY HDACi	FUNCTION	REFERENCE
						
APOPTOSIS	BAK^a^	cell line	SBHA^b^	up-regulation	pro-apoptotic	[54,106,151]
	BAX	cell line	SBHA, NaB	up-regulation	pro-apoptotic	[54,56,106,151]
	BCL-X	cell line	SAHA, SBHA, TSA	down-regulation	anti-apoptotic	[54,106,151,152]
	BID	cell line	SBHA	up-regulation	pro-apoptotic	[151]
	BIM	cell line	SBHA	up-regulation	pro-apoptotic	[54,106,151]
	CASP3	cell line	SBHA	up-regulation	pro-apoptotic	[151]
	CASP8	cell line	SBHA	up-regulation	pro-apoptotic	[151]
	MCL-1	cell line	SBHA	down-regulation	anti-apoptotic	[54,106,151]
	TRAILR1	cell line	SAHA, TSA	up-regulation	pro-apoptotic	[152]
	TRAILR2	cell line	TSA, SAHA	up-regulation	pro-apoptotic	[152]
	XIAP	cell line	SBHA	down-regulation	anti-apoptotic	[54,106,151]
						

CELL CYCLE	CCNA	cell line	TSA	down-regulation	promotes cell cycle progression	[53]
	CCND1	cell line	TSA, VPA	down-regulation	promotes cell cycle progression	[53,114]
	CCND3	cell line	TSA	up-regulation	promotes cell cycle progression	[53]
	CCNE	cell line	TSA	up-regulation	promotes cell cycle progression	[53,153]
	CDKN1A	cell line	LAQ824, VPA, MS-275, NaB, TSA	up-regulation	inhibits cell cycle progression	[53,110,114,154-157]
	CDKN2A	cell line	VPA	up-regulation	inhibits cell cycle progression	[114]
	TP53	cell line	TSA	down-regulation	inhibits cell cycle progression	[53]
						

DNA REPAIR	KU70	cell line	NaB, SAHA, TSA	down-regulation	repairing radiation-induced DNA damages	[119,120]
	KU80	cell line	SAHA	down-regulation	repairing radiation-induced DNA damages	[120]
	KU86	cell line	NaB, TSA	down-regulation	repairing radiation-induced DNA damages	[119]
	RAD50	cell line	SAHA	down-regulation	repairing radiation-induced DNA damages	[120]
						

INVASION/ METASTASIS	CCR7	cell line	TSA	up-regulation	promotes cell migration	[141]
	CXCR4	cell line	TSA	up-regulation	promotes cell migration	[141]
	MMP10	cell line	Apicidin	down-regulation	promotes invasion	[158]
	MMP2	cell line	Apicidin	up-regulation	promotes invasion	[158]
						

SIGNALING	OSMR	cell line	TSA	up-regulation	anti-proliferative signals	[159]
	RAP 1	cell line	FK228	up-regulation	inhibits RAS signaling	[160]
	RARB	cell line	LAQ824	up-regulation	transduces RA signals	[110]

^a^, gene symbol: BAK, BCL2-antagonist/killer; Bax, BCL2-associated X protein; Bid, BH3 interacting domain death agonist; BIM, bcl-2 interacting mediator of cell death; CASP3,caspase-3; CASP8, Caspase 8; CCNA, cyclin A; CCND1, cyclin D1; CCND3, cyclin D3; CCNE, cyclin E; CCNE, cyclin E; CDKN1A, cyclin-dependent kinase inhibitor 1A; LEF-1, lymphoid enhancer factor-1; MCL-1, myeloid cell leukemia sequence 1; MMP2, matrix metallopeptidase 2; MMP10, matrix metallopeptidase 10; OSMR, oncostatin M receptor beta; TP53, tumor protein p53; TRAILR2, TNF-related apoptosis inducing ligand receptor 2; XIAP, X-linked inhibitor of apoptosis.

^b^, HDACi: NaB, sodium butyrate; SAHA, suberoylanilide hydroxamic acid; SBHA, suberic bishydroxamic acid; TSA, tricostatin A; VPA, valproic acid.

Besides histone acetylation status, initial studies have addressed a possible role of aberrant histone methylation in CM. Along this line, CM cells were found to express up-regulated levels of the H3K27 HMT EZH2 [58]. Even though no direct evidence has been provided, over-expression of EZH2 could help CM cells to evade senescence, by suppressing p16^INK4A ^expression, and to invade surrounding tissues, by repressing E-cadherin [59]. Moreover, a reduced expression of the histone demethylase KDM5B, which targets trimethylated H3K4, was found in advanced CM [60]. In A375 CM cells, ectopic expression of KDM5B resulted in the block of the cell cycle in G1/S, accompanied by a significant decrease of DNA replication and cellular proliferation, suggesting this histone demethylase might function as a TSG in CM [60]. These are clearly very preliminary data, which need confirmation in large series of CM tissues and the direct identification of the target genes to define the role of histone methylation in CM biology.

### MicroRNAs

Up to now only limited data is available on miRNA deregulation in CM and on its potential involvement in driving CM tumorigenesis and progression (Table 3). Most of the information were derived from general studies on miRNA expression in tumors of different histotype, among which CM represented a variable proportion (reviewed in [61]). Yet, a CM-specific miRNA profiling study has been recently published, reporting extensive modifications of miRNA patterns in CM as compared to normal melanocytes, as well as identifying modifications of miRNA expression that are potentially associated to the different phases of CM pathogenetic process [62]. Accordingly, Levati *et al *showed that miR-17-5p, miR-18a, miR-20a and miR-92a were over-expressed, while miR-146a, miR-146b, and miR155 were down-regulated in the majority of examined CM cell lines as compared to normal melanocytes. Furthermore, the ectopic expression of miR-155 in CM cells significantly inhibited proliferation and induced apoptosis, though the miRNA target mRNA(s) responsible for this activity have not been identified yet [63]. These upcoming evidences, together with initial studies that have identified the target genes regulated by specific miRNA and their functional effect on tumor biology, strongly suggest that miRNA deregulation might play an important role in CM. Along this line, the transcription factor MITF, a master regulator of melanocytes biology, was found to be regulated by at least 2 different miRNAs, miR-137 and miR-182, which showed opposite alterations. MiR-137 was shown to be down-regulated in selected CM cell lines through the amplification of a Variable Number of Tandem Repeats sequence in its 5' untranslated region, which altered the secondary structure of pri-miR-137, preventing the production of the mature miRNA. This lack of inhibition by miR-137 resulted in the over-expression of MITF in CM cells [64]. On the other hand, miR-182 has been identified as being frequently over-expressed through gene amplification in different CM cell lines and tissues, where it contributed to an increased survival and metastatic potential of neoplastic cells by repressing MITF and FOXO3. Of note, miR-182 appeared to be particularly involved in CM progression, being increasingly over-expressed with evolution from primary to metastatic disease [65]. The interplay between the reported opposing alterations involving miR-137 and miR-182 might represent a molecular mechanism able to orchestrate the complex modulation of MITF expression that appears to be required during CM "lifespan", including its up-regulation in the initial phases of CM tumorigenesis and its down-regulation necessary for CM cells to acquire invasive and metastatic potential. Recent data have suggested that also the expression of the oncogene MET, which is involved in triggering an "invasive growth" program characterized by enhanced cell motility, invasion and resistance to apoptosis, might be regulated by miRNA in CM. Indeed, miR-34b, miR-34c, and miR-199a* were found to negatively regulate MET in cancer cell lines of different histotype, and their exogenous expression in primary CM cell cultures led to a reduced expression of MET and to an impaired MET-mediated motility [66]. Another gene that is crucial for CM progression is integrin β3. Its over-expression is frequently observed in CM and leads to enhanced migratory and invasive potential of neoplastic cells. In this context, Muller *et al *have recently demonstrated that the miRNA let-7a directly regulates integrin β3 by targeting its 3' untranslated region, and that the frequent loss of let-7a in CM is the major cause of integrin β3 over-expression [67]. Another member of the let-7 family, let7-b, was shown to be down-regulated in CM. Let-7b was able to suppress, both directly and indirectly, different cell cycle promoting proteins, including cyclins A, D1, D3 and Cyclin-dependent kinase 4. Thus, it appears that Let-7b is an important negative regulator of CM cell growth and proliferation, and its loss likely plays a crucial role in providing neoplastic cells of the melanocytic lineage with oncogenic properties [68].

**Table 3:** miRNAs altered in human CM

PATHWAY	miRNA	TARGETED GENE	EXPRESSION^a^	SOURCE	REFERENCE
					
APOPTOSIS	miR-15b		up-regulated	tumors and cell lines	[122]
	miR-155	NIK^b ^(?)^c^, SKI (?)	down-regulated	cell lines	[63]
					

CELL CYCLE	miR-193b	cyclin D1	down-regulated	tumors	[161]
	miR 17-92 cluster	c-MYC	up-regulated	cell lines	[62,63]
	miR 106-363 cluster	Rbp1-like (?)	up-regulated	cell lines	[62]
	miR-137	MITF	down-regulated	cell lines	[61,64]
	miR-182	MITF, FOXO3	up-regulated	tumors and cell lines	[61,65]
	miR-221/-222	c-KIT, p27	up-regulated	cell lines	[61,69]
	let-7b	cyclins A, D1, D3, CDK4	down-regulated	tumors	[61,68]
					

INVASION/METASTASIS	miR-373		up-regulated	cell lines	[62]
	miR-137	MITF	down-regulated	cell lines	[61,64]
	miR-182	MITF, FOXO3	up-regulated	tumors and cell lines	[61,65]
	let-7a	ITGB3	down-regulated	cell lines	[61]
	miR-34b	MET	down-regulated	cell lines	[66]
	miR-34c	MET	down-regulated	cell lines	[66]
	miR-199a*	MET	down-regulated	cell lines	[66]
					

TBD^d^	miR-17-5p		up-regulated	tumors and cell lines	[62,63,161]
	miR-146a		down-regulated	cell lines	[63]
	miR-146b		down-regulated	cell lines	[63]
	miR-16		up-regulated	tumors	[161]
	miR-21		up-regulated	tumors	[161]
	miR-22		up-regulated	tumors	[161]
	miR-106b		up-regulated	tumors	[161]
	miR-125b		down-regulated	tumors	[161]
	miR-200c		down-regulated	tumors	[161]
	miR-203		down-regulated	tumors	[161]
	miR-204		down-regulated	tumors	[161]
	miR-205		down-regulated	tumors	[161]
	miR-211		down-regulated	tumors	[161]
	miR-214		down-regulated	tumors	[161]
	miR-768-3p		down-regulated	tumors	[161]
					

^a^, level of expression of miRNAs in CM as compared to that found in normal melanocytes;

^b^, gene symbol: CDK4, cycline dependent kinase 4; FOXO3, forkhead box O3; ITGB3, integrin beta 3; MITF, microphthalmia-associated transcription factor; NIK, nuclear factor-inducing kinase; Rbp1-like, retinoblastoma binding protein 1 like; SKI, v-ski sarcoma viral oncogene homolog;

^c^, predicted target genes that have not been confirmed are indicated with question marks (?);

^d^, TBD: to be determined.

As suggested by the case of let-7b, a peculiar behaviour of miRNA deregulation is that the specific alteration of a single miRNA species may impact the biology of CM cells by concurrently affecting multiple proteins/pathways. Along with this notion, the increased expression of miR-221/222, occurring during CM progression from primary to metastatic disease, was described to down-regulate both p27 and c-KIT, leading to a concomitant increase in cell proliferation and differentiation blockade of CM cells [69].

Lastly, besides mediating epigenetic regulation of gene expression, miRNA can be themselves targets of epigenetic regulation. This is the case, for instance, of miR-34a, which is silenced by aberrant CpG island methylation at its promoter in 43.2% of CM cell lines and 62.5% of primary CM tissues analyzed [70]. However, despite its frequent inactivation in CM, further studies are required to define its role in CM biology.

### Epigenetic drugs

Epigenetic deregulation leads to the concomitant impairment of multiple cellular pathways in CM, and the preservation of this aberrant status is dependent on the retained activity of DNMT and/or HDAC. Thus, both enzymes clearly represent the designated targets for epigenetic intervention in CM, and different inhibitors of their activity have been so far described and utilized in the clinical setting.

#### DNMT inhibitors (for review see [71])

*Nucleoside inhibitors *are represented by different cytosine analogues that function as substrate for DNMT, including 5-azacytidine (Vidaza), 5-AZA-CdR (Dacogen), S110 [72] and zebularine. To exert their activity, nucleoside inhibitors must be incorporated into the genomic DNA of the target cell during the S-phase of the cell cycle. Their methylation by DNMT results in a stable covalent bond between the modified DNA and the enzyme, which is irreversibly inactivated and trapped into the DNA [73,74]. The resulting cellular depletion of DNMT activity leads to the passive demethylation of the neosynthesized DNA [73,74]. These cytidine analogs are the most potent DNA hypomethylating agents available so far, and 5-aza-cytidine and 5-AZA-CdR have been positively used in hematologic malignancies, being also able to induce *in vivo *the expression of specific genes (*P16*, several *CTA*) in both hemopoietic [acute myeloid leukemia (AML), myelodysplastic syndrome (MDS)] [75] and solid tumors (lung cancer, esophageal cancer, malignant pleural mesothelioma) [76]. Their use, however, is associated with a significant cytotoxicity that may be mediated, at least in part, by the triggering of additional cellular events (e.g., genotoxic stress responses), which are not related to hypomethylation but strictly inherent with the mode of action of these drugs.

*Non nucleoside inhibitors *directly block the DNMT activity without needing to be incorporated into the DNA, thus are not expected to give toxicity related to the covalent trapping of the enzyme. Within this class, different compounds have been associated with different modalities of action: i) procaine and procainamide interfere with the binding of DNMT to the substrate DNA; ii) (-)-epigallocatechin-3-gallate and RG108 bind and block the DNMT catalytic site; iii) the MG98 antisense oligonucleotide triggers degradation of DNMT mRNA. Of these, MG98 has undergone clinical evaluation in Phase I and II trials conducted in patients with solid (colorectal, cervix, esophagus, lung, ovary, renal) or hematopoietic (AML, MDS) malignancies, but failed to demonstrate any significant clinical activity [77-79].

#### HDAC inhibitors

HDACi (for review see [80]) can be classified into different classes based on their chemical structure: short chain fatty acids (e.g., butyrate, valproic acid), hydroxamic acids [e.g., TSA, suberoylanilide hydroxamic acid (SAHA, vorinostat, ZOLINZA), suberic bishydroxamic acid (SBHA), PXD101 (Belinostat), LAQ824], cyclic tetrapeptides [e.g., trapoxin A, apicidin, depsipeptide (romidepsin)], benzamides [e.g., MS-275 (SNDX-275), CI-994]. Most of them are suggested to act by blocking the Zn^2+ ^containing catalytic site of HDAC. HDACi cause accumulation of acetylated histones into the nucleosomes, resulting in a more accessible and transcriptionally active chromatin structure. This activity has been linked to their ability to revert aberrant epigenetic marks in human neoplasia. Histones, however, are not the only targets for HDAC, and the comprehensive effects of HDACi may result, at least in part, from mechanisms that are unrelated to direct chromatin remodelling.

### Clinical translation

#### Epigenetic therapies

Treatment of CM cells with epigenetic drugs has clearly demonstrated to have pleiotropic effects sustained by the reactivation of different pathways that became aberrantly inactivated during neoplastic transformation of melanocytes [81]. From a therapeutic perspective, it would thus be tempting to combine epigenetic intervention with conventional and/or innovative therapeutic approaches that would take specific advantages from the epigenetically-restored functionality of deregulated pathways. To this end, despite different epigenetic drugs have already been used extensively in the clinic (Table 4) [82-84], and recent *in vitro *and *in vivo *evidences show that these drugs preferentially target neoplastic cells [85-88], additional pre-clinical studies are likely required to more precisely define their effects on normal cells and to predict their safety for patients. Along this line, validation of recent investigations, reporting potential molecular markers of *in vitro *sensitivity/resistance to epigenetic drugs [89], is required prior to their clinical application for selecting patients who will benefit most from epigenetic treatment.

**Table 4:** Ongoing clinical trials with epigenetic drugs in CM patients

	Molecule (commercial name)	Tumor	Phase	Combination	Identifier^a^
					
**inhibitors of DNMT**	5-azacytidine (Vidaza)	Melanoma (Skin)	I	Recombinant Interferon α-2b	NCT00398450
		Kidney Cancer, Melanoma (Skin)	I	Recombinant Interferon α-2b	NCT00217542
					
	5-Aza-2'-deoxycytidine (Dacogen, Decitabine)	Melanoma	I, II	Pegylated Interferon α-2b	NCT00791271
		Metastatic Melanoma	I, II	Temozolomide, Panobinostat	NCT00925132
		Melanoma	I, II	Pegylated Interferon α-2b	NCT00791271
		Melanoma	I, II	Temozolomide	NCT00715793
					
					

**inhibitors of HDAC**	Valproic acid (Depakote, Depakote ER, Depakene, Depacon, Stavzor)	Melanoma	I, II	Karenitecin	NCT00358319
					
	FR901228 (Romidepsin)	Intraocular Melanoma, Unresectable stage III or stage IV Melanoma	II		NCT00104884
					
	MS-275 (Entinostat, SNDX-275, BAY86-5274)	Melanoma	II		NCT00185302
					
	Suberoylanilide hydroxamic acid, SAHA (Vorinostat, Zolinza)	NSCLC, Pancreatic Cancer, Melanoma, Lymphoma	I	Protesome inhibitor NPI-0052	NCT00667082
		Intraocular Melanoma, Metastatic or Unresectable Melanoma	II		NCT00121225
					

^a^, identifier of the trial as retrieved from: http://www.clinicaltrials.gov.

A growing body of experimental evidences identifies a potent immunomodulatory activity of epigenetic drugs. In fact, 5-AZA-CdR was able to induce or to up-regulate the expression of CTA in CM cells both *in vitro *and *in vivo*, allowing their recognition by CTA-specific cytotoxic T lymphocytes (CTL), and generating high titre anti-CTA antibodies *in vivo *[47,85,90-92]. Moreover, 5-AZA-CdR was able to revert the constitutively heterogeneous intratumoral expression of CTA, allowing an homogeneous intratumoral targeting of CM cells by CTA-specific CTL [49]. CTA do not appear to be the sole immunotherapeutic targets modulated by hypomethylating treatment, since the High Molecular Weight-Melanoma Associated Antigen was recently reported to be re-activated by 5-AZA-CdR in CM cells [93], and the tyrosinase-related protein 2 was reactivated by the hypomethylating treatment in B16 murine CM cells [94]. Besides tumor antigens, 5-AZA-CdR has a broader immunomodulatory activity, being able to concomitantly up-regulate molecules that are essential for the presentation of immunogenic peptides to immune cells, and for the recognition and cytotoxicity of CM cells by effector T-cells: 5-AZA-CdR up-regulated HLA class I antigens and accessory/co-stimulatory molecules (e.g., CD54, CD58), resulting *per se *in an increased recognition of CM cells by antigen-specific CTL [90,95-97]. The ability of 5-AZA-CdR to re-establish the expression of different molecules required by CM cells to undergo immune-triggered apoptosis (TRAILR1, XAF1, RASSF1A, caspase 8), represents a further important effect that may ensure an efficient immune eradication of neoplastic cells [41,98-100]. Nevertheless, this effect might not be taken as granted, since Liu *et al *have recently reported that demethylating agents may also up-regulate TRAIL decoy receptors that antagonize TRAIL-induced apoptosis [101]. In this epigenetic immunomodulatory scenario, HDACi may contribute with their demonstrated ability to up-regulate different molecules, including: FAS, the melanoma antigen gp100, molecules involved in antigen processing and presentation (MHC class I and II antigens, TAP1, TAP2, LMP2, LMP7 and Tapasin), and the co-stimulatory molecules CD40 and B7-1/2 in B16 murine CM cells [102-105]. These modulations of the antigenic profile of CM cells associated with a significant increase in direct presentation of MHC class I- and II-restricted peptides by HDACi-treated B16 cells, and to their increased apoptosis following FASL treatment [103-105]. Similarly, human CM cells underwent increased apoptosis upon the synergistic action of TRAIL and the HDACi SBHA [106]. The above reported immunologic modulations, which also include an increased antigen cross presentation *in vivo*, likely explain the observation that vaccination of mice with HDACi-treated B16 cells induced specific anti-tumor immunity that was able to control the growth of established B16 tumors (therapeutic vaccination) and to prevent tumor take by subsequent challenge with B16 CM cells (prophylactic vaccination) [104]. Altogether, the information above provide a strong scientific background to translate treatments combining epigenetic drugs and immunotherapies into clinical development. Along this line, Kozar et al demonstrated that IL12 immunotherapy improves the antitumor effectiveness of 5-AZA-CdR in B16 CM model in mice, and that this synergism requires the presence of CD4+ and CD8+ T lymphocytes [107]. Moreover, *in vivo *administration of HDACi has proven particularly effective in enhancing the antitumor activity of adoptively transferred antigen- or tumor-specific T cells in mice, through a coordinate action on both tumor and T cells [105,108]. Indeed, besides the phenotypic modulations induced on CM cells, the immunotherapeutic activity of HDACi appeared to also rely on their ability to: i) provide a proliferative advantage to adoptively transferred cells, mediated by a preferential depletion of naïve endogenous lymphocytes in the recipient mice; ii) improve the functionality of the adoptively transferred lymphocytes, which showed a higher cytotoxic potential *in vivo *[108]. In this context, Gollob et al have recently performed a phase I trial of 5-AZA-CdR plus high-dose IL2 in CM and renal carcinoma patients, demonstrating that the combination is well-tolerated and that 5-AZA-CdR may enhance the activity of IL2 [109]. In light of these promising data, additional epigenetic-based immunotherapy studies are likely to be expected in the next future (Table 4).

Keeping on in the field of biologic therapies, 5-AZA-CdR or HDACi in association with RA demonstrated to be able to re-express RAR-β2. Combined treatment resulted in a reduced clonogenicity and in an impaired growth of CM cells *in vivo*, suggesting for the potential clinical effectiveness of this therapeutic association [39,110]. Recent data, however, showed that the expression of PRAME may prevent the re-activation of RAR-β2 by epigenetic drugs. This observation led to the patenting of a therapeutic strategy that foresees treatment with an inhibitor of PRAME in conjunction, or prior, to HDACi and RA therapy (Table 5, Pat. n: CA2553886). Tumor angiogenesis has become an attractive therapeutic target in different malignancies, though it is now clear that the most efficient clinical use of anti-angiogenic drugs is through combination therapies. In this respect, epigenetic drugs might represent appealing combination partners in light of the recent demonstration that 5-AZA-CdR, zebularine and TSA counteract the pro-angiogenic stimuli mediated by tumor conditioned medium, finally resulting in a reduced vessel formation in different tumor models [111].

**Table 5:** Published patents on CM epigenetics^a^

TITLE	PATENT NUMBER	PUBLICATION DATE
		
ADMINISTRATION OF AN INHIBITOR OF HDAC AND AN HMT INHIBITOR	WO2009126537	15/10/2009
USE OF METHYLATION STATUS OF MINT LOCI AND TUMOR-RELATED GENES AS A MARKER FOR MELANOMA AND BREAST CANCER	WO2009086472	09/07/2009
GENE METHYLATION IN DIAGNOSIS OF MELANOMA	US2009170083	02/07/2009
ADMINISTRATION OF AN INHIBITOR OF HDAC	WO2009067500	28/05/2009
MARKERS FOR MELANOMA	US2009093424	09/04/2009
USE OF HDAC INHIBITORS FOR THE TREATMENT OF MELANOMA	CA2684114	20/11/2008
UTILITY OF HIGH MOLECULAR WEIGHT MELANOMA ASSOCIATED ANTIGEN IN DIAGNOSIS AND TREATMENT OF CANCER	WO2008121125	09/10/2008
INHIBITORS OF DNA METHYLATION IN TUMOR CELLS	US2008138329	12/06/2008
METHODS AND PRODUCTS FOR DIAGNOSING CANCER	WO2008066878	05/06/2008
UTILITY OF HIGH MOLECULAR WEIGHT MELANOMA ASSOCIATED ANTIGEN IN DIAGNOSIS AND TREATMENT OF CANCER	WO2007123697	01/11/2007
MARKERS FOR MELANOMA	EP1840227	03/10/2007
MARKERS FOR MELANOMA	WO2006092610	08/09/2006
COMBINED USE OF PRAME INHIBITORS AND HDAC INHIBITORS	CA2553886	18/08/2005
USE OF BAGE (B MELANOMA ANTIGENS) LOCI AS TUMOUR MARKERS	WO2004101822	25/11/2004

^a^, as retrieved from database search on: http://ep.espacenet.com

In addition to biologic therapies, epigenetic drugs are expected to be successful also in combination with standard cancer chemo- and radio-therapeutic approaches (Table 4). In fact, re-expression/up-regulation of caspase 8 and/or of APAF-1 by 5-AZA-CdR may sensitize CM cells to apoptosis induced by adriamycin, cisplatinum, doxorubicin, and etoposide [55,100]. Furthermore, resistance of tumor cells to alkylating drugs is associated to an increased expression of MGMT, which repairs the DNA alterations induced by these drugs. Although surprising, recent reports indicate an association between MGMT re-expression in CM cells and intragenic hypermethylation around exon 3 [112,113]. Consistently, 5-AZA-CdR treatment down-regulated MGMT activity in CM cells, partly reverting their sensitivity to alkylating drugs [112,113]. As far as HDACi, these agents were demonstrated to be able to sensitize CM cells to apoptosis induced by cisplatinum and topoisomerase inhibitors [114,115]. These data led to the development of different clinical trials with HDACi alone or combined with chemo or chemoimmunotherapeutic regimens in CM (Table 4)[116-118]. Results were promising, being the combination generally well-tolerated and frequently associated with stabilization of the disease [116,118]. Nevertheless, Rocca *et al *reported that combination of valproic acid and dacarbazine plus interferon-α resulted in an increased toxicity and no superior clinical efficacy as compared to the standard therapy in patients with advanced CM [117]. Thus, it appears that the clinical efficacy of HDACi combinations strictly depends on the setting in which they are utilized. Besides chemotherapeutic drugs, HDACi were demonstrated to synergize also with radiation, reducing the clonogenic survival of CM cells. The radiosensitizing activity of HDACi seems to be related to their ability to sensitize CM cells to radiation-induced apoptosis and to impair the ability of CM cells to repair radiation-induced DNA damages (down-regulation of the repair proteins Ku70, Ku80, Ku86 and Rad50) [119,120]. Altogether, the above reported data strongly support the future development of combined epigenetic chemo/radiotherapies that might overcome the currently limited efficacy of conventional therapies in CM.

#### Prognostic and predictive epigenetic markers

The epigenetic alterations found in CM may be exploited also to define new markers for diagnosis or prediction of disease outcome and/or response to therapy. Along this line, different patents exist promoting the analysis of the methylation status of selected genes (e.g., *TSPY1, CYBA, MX1, MT2A, RPL37A, HSPB1, FABP5, BAGE*) as additional diagnostic tool for CM, which might also predict the likelihood of metastatic spreading (Table 5). Upcoming literature data have provided some initial validation on the potential prognostic role of epigenetic alterations in CM. By analyzing 230 primary CM, Lahtz *et al *demonstrated that *PTEN *methylation in CM tissues is an independent predictor of impaired patient survival, though its prognostic relevance was not superior to tumor thickness and ulceration [121]. On the other hand, aberrant hypermethylation of *MINT31 *locus was recently found to be a significant predictor of improved overall survival in stage III CM patients. Nevertheless, the small number of patients examined in this study requires further validations to draw general conclusions [42]. Besides DNA methylation markers, initial data are suggesting that also alterations in miRNAs expression might have a prognostic role in CM. Indeed, a recent paper described a significant association between an up-regulated expression of miRNA miR-15b in primary CM lesions and a poor recurrence-free and overall survival of patients [122]. In line with these data, different studies have investigated the methylation status of several genes in sera of CM patients, with the aim to provide reliable soluble prognostic epigenetic markers that could be easily assayable in the routine laboratory. Albeit conducted on a small number of patients, the results of these initial studies are encouraging: i) serum *ER-α *methylation in stage IV CM patients was a negative predictor of overall and progression-free survival in patients treated with biochemotherapy (dacarbazine or temozolomide, cisplatinum, vinblastine, interferon-α2b, IL2, and tamoxifen) [123]; ii) serum *RASSF1A *methylation inversely correlated with overall survival and biochemotherapy response in CM patients [124]. Most recently, methylation of the *p73 *gene was found to be associated to an increased sensitivity of CM cells to alkylating agents *in vitro *[125], suggesting it as a potential marker to be assayed in patients to predict response to therapy. Along this line, *MGMT *promoter methylation has been evaluated in CM patients undergoing therapy with the alkylating agent temozolomide. A trend towards a positive correlation was found between *MGMT *promoter methylation level ≥ 25% and the achievement of partial clinical responses to the drug, suggesting further evaluations in clinical trials [126]. The development of new diagnostic or prognostic epigenetic tools is clearly an exploding field in the translational research of CM, and it might also take advantage of the recent identification of genes that are hypermethylated in virtually all CM lesions (e.g., *QPCT, LXN*) [21] (Table 5).

## Conclusion

Epigenetic alterations clearly play a major role in CM biology, and epigenetics of CM is a rapidly growing field that promises appealing therapeutic and diagnostic developments. The upcoming availability of next-generation sequencing technologies, at increasingly affordable costs, is expected to allow defining the complete epigenome of CM in the near future. This in-depth knowledge will provide a full understanding of the biological aspects altered by epigenetic modifications during CM tumorigenesis and progression, granting new therapeutic targets, as well as more effective prognostic and/or predictive markers to be implemented in the daily clinical management of CM patients. Concomitantly, new generation epigenetic drugs can be expected to be developed to achieve reduced systemic toxicities, higher bioavailability, and a more specific epigenetic effect. Concerning the latter aspect, it should be kept in mind that: i) the highly effective nucleoside inhibitors of DNMT may trigger a DNA hypomethylation-unrelated cytotoxic response caused by covalent trapping of DNMT into DNA; ii) HDACi induce hyperacetylation of many non-histone proteins, resulting in cellular effects that may not depend on epigenetic regulation of gene expression. One clear future direction is therefore to find more specific epigenetic remodelling agents. Along this path, the recent definition of a three-dimensional model for the catalytic site of the human DNMT1 allowed to select *in silico *the small molecule RG108 as a specific inhibitor of DNMT1. RG108 was then demonstrated to inhibit the activity of purified DNMT *in vitro *and to hypomethylate tumor suppressor genes in human neoplastic cell lines, yet having a negligible toxicity as compared to nucleoside analogs [127]. This encouraging result prompts further efforts in designing new drugs with specific epigenetic remodelling properties, which could represent even more suitable agents to be implemented in epigenetic therapies in CM patients.

## Competing interests

The authors declare that they have no competing interests.

## Authors' contributions

LS and MM both conceived of the manuscript, and participated in its design and coordination. All authors made intellectual contributions and participated in the acquisition, analysis and interpretation of literature data, have been involved in drafting the manuscript and approved the final manuscript.
